# Latent Profiles of Academic Anxiety and Working Memory in Adults with Varying Dyslexia-Related Risk

**DOI:** 10.3390/jintelligence14070114

**Published:** 2026-06-23

**Authors:** Liming Zhang, Lijuan Kou, Jingyu Yang, Xinhui Ma, Ke Zhang

**Affiliations:** 1 School of Education, Huainan Normal University, 238 Dongshan West Road, Tianjia’an District, Huainan 232038, China; 18392035681@163.com; 2 Institute of Brain and Psychological Sciences, Sichuan Normal University, No. 5, Jing’ an Road, Jinjiang District, Chengdu 610066, China; koukou3212@126.com; 3 School of Psychology, Shaanxi Normal University, 199 Chang’an South Road, Yanta District, Xi’an 710062, China; yangjingyu1@stu.xawl.edu.cn

**Keywords:** dyslexia-related risk, academic anxiety, working memory, reading anxiety, mathematics anxiety, latent profile analysis

## Abstract

This study examined whether adults with varying levels of dyslexia-related risk show distinct profiles of academic anxiety and working memory functioning. Dyslexia-related risk was assessed with the corrected Chinese Adult Reading History Questionnaire (Chinese-ARHQ). Latent profile analysis was conducted using reading anxiety, mathematics anxiety, a digit-span working memory composite, and operation span as profile indicators. Corrected Chinese-ARHQ scores were then compared across the retained profiles as an external variable, and a sensitivity analysis was conducted in the elevated dyslexia-related risk subsample defined by Chinese-ARHQ scores above 0.36. A four-profile solution was retained: Low Anxiety-Average Cognition, High Anxiety-High Cognition, Very Low Verbal Working Memory, and High Anxiety-Low Cognition. The coexistence of high-anxiety profiles with different levels of cognitive performance suggests that, within the specific profile distribution of this sample, elevated academic anxiety was not consistently accompanied by uniformly lower working memory performance. Corrected Chinese-ARHQ scores did not differ significantly across profiles, suggesting that the identified emotion-cognition patterns were not closely aligned with retrospective reading-difficulty severity in this sample. Overall, the findings provide preliminary evidence that current academic anxiety and working memory performance may form distinguishable configurations among adults with varying dyslexia-related risk, while also highlighting the need for replication with independent and clinically characterized samples.

## 1. Introduction

Developmental dyslexia is commonly associated with persistent difficulties in word recognition, decoding, spelling, and reading fluency. Although dyslexia is often identified during childhood, reading-related difficulties may continue into adulthood and can affect academic activities, occupational functioning, and psychological well-being (Snowling et al., 2020). Adults with a history of reading difficulties may continue to experience reading tasks as effortful, particularly when they involve time pressure, public performance, or evaluation. Importantly, some adults without a formal diagnosis may nevertheless report reading-related risk characteristics, such as a history of reading difficulties, reduced reading efficiency, effortful reading, or difficulties in reading comprehension (Reis et al., 2020; He et al., 2025). Although dyslexia-related risk should not be equated with a clinical diagnosis of developmental dyslexia, it may still reflect a form of developmental vulnerability shaped by interactions among literacy experience, cognitive resources, emotional responses, and adaptation (Moojen et al., 2020; Reis et al., 2020). Accordingly, the present study focused on dyslexia-related risk rather than clinically diagnosed developmental dyslexia. In the present study, dyslexia-related risk was indexed by the Chinese Adult Reading History Questionnaire and was therefore treated as a screening-based indicator of retrospective reading-difficulty history rather than as a diagnostic category.

Anxiety is one emotional factor that appears especially relevant in the context of reading difficulty. Individuals with reading difficulties may report heightened anxiety in literacy-related situations, such as reading aloud, completing reading assignments, or being evaluated on reading performance (Carroll & Iles, 2006; Francis et al., 2019). Such anxiety may also extend beyond reading contexts. Repeated experiences of difficulty, greater task effort, and negative academic self-appraisal could plausibly contribute to anxiety in other symbolically demanding academic domains, including mathematics (Brumariu et al., 2023; Xiao et al., 2022). Individuals with dyslexia or elevated risk of dyslexia may be more vulnerable to negative emotional experiences in academic contexts, including anxiety related to reading and, in some cases, mathematics. Previous evidence suggests that students with dyslexia can report higher levels of mathematics anxiety than their peers without dyslexia, although this association should not be interpreted as indicating that mathematics anxiety is inherent to dyslexia (Jordan et al., 2014). Mathematics anxiety is often experienced when individuals are required to understand, manipulate, or apply mathematical information; importantly, many mathematical tasks, particularly word problems, also depend on reading comprehension and the integration of verbal information (Dowker et al., 2016; Lai et al., 2015). Recent work further suggests that arithmetic and reading skills may jointly contribute to the relation between mathematics anxiety, reading anxiety, and word-problem performance (Jaffe & Bolger, 2023; Tal et al., 2025). More broadly, reading anxiety and mathematics anxiety appear to be related but separable forms of academic anxiety, indicating that they may co-occur in some individuals while still reflecting partly domain-specific experiences (Edwards et al., 2023; Sasanguie et al., 2024). One possible mechanism underlying this co-occurrence is working memory: anxiety-related worry may consume cognitive resources needed for maintaining and manipulating task-relevant information, and working memory has been implicated in both reading comprehension and mathematical problem solving (Ashcraft & Krause, 2007; Moran, 2016; Peng et al., 2018). Therefore, in adults at elevated risk of dyslexia, the co-occurrence of reading and mathematics anxiety may be partially reflected in working-memory-related difficulties, although the direction and strength of these associations require further empirical examination.

Despite increasing attention to anxiety and broader internalizing problems among individuals with learning difficulties, several issues remain unresolved (Vieira et al., 2024). First, many studies have relied on variable-centered approaches that estimate average associations among dyslexia-related risk, anxiety, and cognitive performance. Although such approaches are useful, they may obscure meaningful subgroups of individuals who show different configurations of emotional, motivational, and cognitive characteristics. Person-centered approaches, such as latent profile analysis, are therefore useful for identifying heterogeneous profiles within a broader population (Broks et al., 2024; Matt & Michael, 2018). Second, studies conducted in broad community or student samples may have difficulty separating the possible roles of developmental reading history, anxiety, and current cognitive functioning, particularly because adult dyslexia-risk measures are often screening tools rather than diagnostic evaluations. Thus, it remains important to examine whether adults with comparable levels of dyslexia-related risk show heterogeneous emotion-cognition patterns.

For example, some adults with learning difficulties may experience heightened anxiety without showing a straightforward association between anxiety and working memory performance. A study of Chinese undergraduates with and without specific learning disabilities found that the direct predictive effects of state anxiety and working memory on academic performance were not significant among students with SLD; instead, the anxiety-performance association became evident mainly among students with relatively poorer working memory (Wang et al., 2025). Similarly, Chow et al. (2021) found that reading anxiety and verbal working memory independently predicted ESL reading comprehension in Chinese undergraduates, but the interaction of reading anxiety and working memory was not significant (Chow et al., 2021). These findings suggest that the relation between anxiety and working memory may not be uniform across individuals, and that some adults with elevated dyslexia-related risk may show anxiety-related difficulties without parallel impairments in working memory. This provides further rationale for using a person-centered approach to identify heterogeneous emotion-cognition profiles rather than assuming a single average pattern across all individuals.

The Adult Reading History Questionnaire (ARHQ) provides a useful indicator of retrospective reading-difficulty history. Rather than directly measuring current reading performance, the ARHQ assesses developmental indicators of literacy-related difficulty, making it suitable for identifying adults with probable dyslexia-related risk (Lefly & Pennington, 2000; Welcome & Meza, 2019). The Chinese version of the ARHQ has recently been validated, with a cutoff score of 0.36 showing satisfactory screening characteristics (He et al., 2025). However, ARHQ scores should be interpreted as a distal risk indicator rather than as a complete description of current cognitive or emotional functioning. A higher ARHQ score may suggest a stronger history of reading difficulty, but it does not necessarily determine a person’s current working memory performance or academic anxiety profile (Eckert et al., 2025).

The present study therefore used a person-centered approach to examine profiles of academic anxiety and working memory in an adult undergraduate sample with varying levels of dyslexia-related risk. Reading anxiety, mathematics anxiety, digit-span working memory, and operation span were used as profile indicators in latent profile analysis (LPA). Corrected Chinese-ARHQ scores were then compared across profiles as an external variable to examine whether the profiles differed in retrospective reading-difficulty history. We expected to observe heterogeneous emotion-cognition profiles rather than a single pattern in which higher anxiety uniformly corresponded to lower cognitive performance. Because the ARHQ was treated as a background risk indicator rather than a profile-defining variable, we examined whether profile differences were mirrored by ARHQ differences without assuming that ARHQ severity would fully account for the identified profiles.

## 2. Materials and Methods

### 2.1. Participants

Participants were undergraduate students recruited from Huainan Normal University through campus-based convenience sampling. Eligible participants were native Chinese-speaking adults aged 18 to 25 years and reported no history of neurological disorders. The final sample included 550 undergraduate students (323 women; mean age = 19.5 years). Dyslexia-related risk was measured using the corrected Chinese Adult Reading History Questionnaire (Chinese-ARHQ). Corrected Chinese-ARHQ scores ranged from 0.239 to 0.902, indicating variability in retrospective reading-difficulty history. Using the validated cutoff score of 0.36 reported by He et al. (2025) 500 participants (90.91%) scored above the cutoff and were classified as showing elevated dyslexia-related risk, whereas 50 participants (9.09%) scored at or below the cutoff and were classified as lower risk. The full sample was therefore treated as a heterogeneous undergraduate sample with varying levels of dyslexia-related risk, with the elevated-risk subsample used for a sensitivity analysis.

The recruitment announcement described the study as an investigation of reading-ability risk. Thus, although participants were not selected on the basis of Chinese-ARHQ scores, the recruitment wording may have increased participation among students with reading-related or academic difficulties. In addition, because participants were recruited from a single regional public university, the sample may not represent students from other institutional contexts. Therefore, the high proportion of participants above the Chinese-ARHQ cutoff should be interpreted as a characteristic of the recruited convenience sample rather than as a prevalence estimate for Chinese undergraduates or for the broader population of 18- to 25-year-old students.

### 2.2. Measures

#### 2.2.1. Chinese Adult Reading History Questionnaire (Chinese-ARHQ)

Dyslexia-related risk was assessed retrospectively using the 23-item Chinese-ARHQ (He et al., 2025). The questionnaire assesses current reading experiences, dyslexia-related symptoms, and memory-related difficulties. Participants responded on a 5-point scale from 0 to 4. Following the standardized scoring procedure, a total score was calculated by dividing the sum of all item scores by the maximum possible score of 92, yielding a score from 0 to 1. Higher scores indicate a stronger retrospective history of reading difficulty. In the present study, ARHQ scores were used to describe dyslexia-related risk, to classify the elevated-risk subsample, and to compare risk levels across the identified profiles. The Chinese-ARHQ has shown good test–retest reliability and internal consistency, with satisfactory sensitivity and specificity at the cutoff score of 0.36 (He et al., 2025). In the present sample, internal consistency was high (Cronbach’s alpha = 0.86). ARHQ yielded a more complex six-factor solution (KMO = 0.820, Bartlett’s χ^2^(253) = 5017.17, *p* < 0.001), accounting for 62.40% of the total variance. The emergent factors mapped onto theoretically meaningful dimensions, including reading attitudes, childhood reading difficulties, and current reading ability, thereby supporting the multidimensional nature of the construct.

#### 2.2.2. Reading Anxiety

Reading-related anxiety was assessed using a 13-item Likert-type scale adapted and expanded from the Reading Anxiety in College Students (RACS) scale and related reading-affect items reported by Edwards et al. (2023). The original RACS was designed to assess reading anxiety in college students. In the present study, the scale included 10 items assessing anxiety, discomfort, avoidance, nervousness, and feelings of being overwhelmed in reading-related situations. Three additional items were included to capture reading-related self-perception and reading enjoyment: perceived difficulty reading well or quickly despite effort, perceived poorer reading ability relative to others, and enjoyment of reading alone. Participants responded on a 5-point Likert-type scale. The reading enjoyment item was reverse-scored so that higher total scores consistently reflected greater reading-related anxiety or more negative reading-related affect. The total score was calculated by summing the 13 items. In the present sample, internal consistency was high (Cronbach’s alpha = 0.919). The reading anxiety scale yielded a two-factor solution (KMO = 0.925, Bartlett’s χ^2^(78) = 5115.09, *p* < 0.001), accounting for 66.31% of the total variance, with all factor loadings exceeding 0.60.

#### 2.2.3. Math Anxiety

Math anxiety was assessed using a scale adapted from the Mathematics Anxiety Rating Scale (Suinn et al., 1972; Suinn & Edwards, 1982). Items assess tension and apprehension experienced during numerical operations, mathematics examinations, and everyday situations involving calculation under pressure. Higher scores indicate greater mathematics-related anxiety. In the present sample, internal consistency was high (Cronbach’s alpha = 0.967). The math anxiety scale (24 items) produced a two-factor structure (KMO = 0.966, Bartlett’s χ^2^(276) = 11,870.32, *p* < 0.001) that explained 67.30% of the variance, with all loadings surpassing 0.50. Together, these results indicate that both anxiety measures capture internally coherent, well-differentiated dimensions of domain-specific anxiety.

#### 2.2.4. Digit Span Tasks

Digit Span Forward and Digit Span Backward tasks were adapted from the Wechsler Adult Intelligence Scale (Ostrosky-Solís & Lozano, 2006; Valentine et al., 2020). In Digit Span Forward, participants recalled increasingly long digit sequences in the same order, providing an index of verbal short-term memory. In Digit Span Backward, participants recalled digit sequences in reverse order, which requires both storage and mental manipulation. For both tasks, the total number of correctly recalled sequences was recorded. Internal consistency was high for Digit Span Forward (Cronbach’s alpha = 0.854) and Digit Span Backward (Cronbach’s alpha = 0.898). For the digit span tasks, separate EFAs were conducted in light of their distinct cognitive demands. Digit Span Forward (9 trials) produced a two-factor structure (KMO = 0.896, χ^2^(36) = 1815.40, *p* < 0.001) explaining 60.03% of the variance, with the first factor loading on easier trials (items 1–6) and the second on more challenging trials (items 7–9). Digit Span Backward (10 trials) similarly yielded a two-factor solution (KMO = 0.927, χ^2^(45) = 3246.24, *p* < 0.001) that accounted for 68.21% of the variance. A summed digit span score was used as the verbal working memory indicator.

#### 2.2.5. Operation Span (OSPAN)

The OSPAN task was administered using a computerized E-Prime program to assess complex working memory capacity. Participants solved arithmetic verification problems while remembering target letters. After sets of two to seven equation-letter pairs, participants recalled the letters in serial order (Unsworth et al., 2005). To reduce the likelihood that participants prioritized memory over processing, a minimum accuracy threshold of 85% on the arithmetic problems was required. The final score was calculated using the partial-credit unit method, representing the number of letters recalled in the correct position across trials. The task followed the general paradigm introduced by (Turner & Engle, 1989).

#### 2.2.6. Data Analysis

Full Information Maximum Likelihood was used to estimate models in the presence of missing data (Enders, 2001). Analyses were conducted in two steps. First, latent profile analysis was performed in Mplus 8.2 to identify subgroups characterized by similar patterns of academic anxiety and working memory performance. Four standardized indicators were entered into the LPA: reading anxiety, mathematics anxiety, summed digit span working memory, and operation span. Standardization allowed the indicators to be compared on a common metric.

Models with three to six profiles were compared using multiple criteria, including the Akaike Information Criterion (AIC), Bayesian Information Criterion (BIC), sample-size adjusted BIC, entropy, the Vuong–Lo–Mendell–Rubin likelihood ratio test (VLMR-LRT), profile size, and substantive interpretability (Nylund et al., 2007). Lower information criteria indicate better relative fit, whereas higher entropy indicates clearer classification. The VLMR-LRT compares a *k*-profile model with a *k* − 1 profile model. Profile solutions were also evaluated for stability and interpretability, and profiles representing fewer than approximately 5% of the sample were treated cautiously (Tein et al., 2013).

After the profile solution was selected, corrected Chinese-ARHQ scores were compared across latent profiles as an external variable. ARHQ scores were not included as profile indicators. A one-way analysis of variance (ANOVA) was conducted with profile membership as the independent variable and corrected ARHQ score as the dependent variable. The proportion of participants above the Chinese-ARHQ cutoff was also examined descriptively. As a sensitivity analysis, the LPA was repeated among participants whose corrected Chinese-ARHQ scores exceeded the validated cutoff of 0.36. The same four indicators and the same model-selection criteria were used. Because this subsample was drawn from the full sample, the analysis was interpreted as a within-sample sensitivity analysis rather than as an independent replication.

## 3. Results

### 3.1. Descriptive Statistics and Preliminary Analyses

Table 1 presents descriptive statistics and bivariate correlations for the focal variables in the full sample (*N* = 550). Reading anxiety and mathematics anxiety were moderately and positively correlated (r = 0.564, *p* < 0.001). The working memory indicators were also positively intercorrelated (rs = 0.127 to 0.659, *p*s < 0.01 to <0.001), suggesting related but non-identical aspects of cognitive functioning. ARHQ scores showed small positive correlations with reading anxiety and mathematics anxiety but were not significantly correlated with the working memory indicators.

#### 3.1.1. Identification of Emotion-Cognition Profiles via LPA

The present study used standardized scores to describe the emotion–cognition profiles identified by LPA. The fit indices for the 3- to 6-profile solutions are presented in Table 2. In terms of information criteria, AIC, BIC, and sample-size adjusted BIC continued to decrease as the number of profiles increased. Although the 5- and 6-profile solutions showed slightly better fit on these indices, model selection was not based on fit statistics alone. The VLMR-LRT indicated that the 4-profile solution provided a significantly better fit than the 3-profile solution, *p* = 0.003, and the 5-profile solution also showed improvement over the 4-profile solution, *p* = 0.023. However, the 6-profile solution was not significantly better than the 5-profile solution according to the same criterion, *p* = 0.064. In addition, the 5-profile solution included a very small class comprising only 2.78% of the sample, which raised concerns regarding the stability and interpretability of the solution. By contrast, the 4-profile solution showed acceptable classification accuracy (entropy = 0.83), and all profiles accounted for more than 10% of the total sample, supporting meaningful interpretation.

In addition to the fit indices, the substantive meaning of each profile was examined based on the four indicators used to generate the profiles, namely reading anxiety, math anxiety, working memory, and operation span. The sample size of each latent profile was also considered to determine whether each class was sufficiently large for interpretation. In the 4-profile solution, the profile sizes were 117, 295, 57, and 80, respectively, all exceeding 5% of the total sample. Taken together, considering statistical fit, classification quality, profile size, and theoretical interpretability, the 4-profile solution was retained as the optimal model.

**Figure 1:**
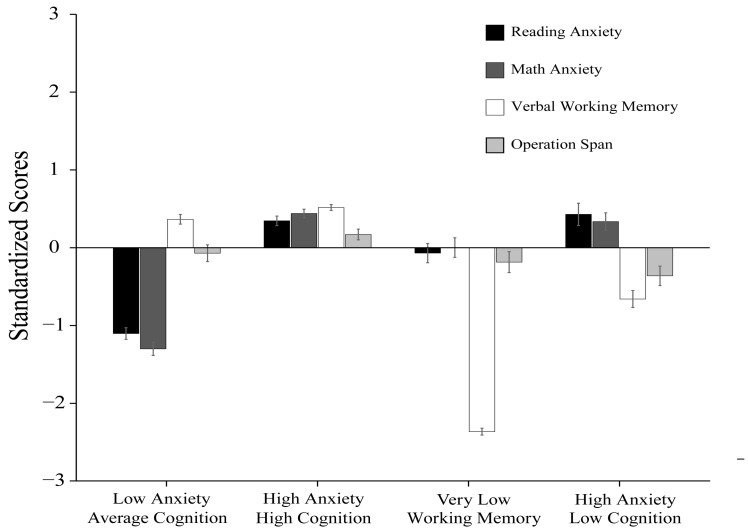
Standardized scores of emotional and cognitive indicators across the four profiles. Error bars represent standard errors.

Table 3 presents the characteristics of each profile based on the standardized scores of reading anxiety, math anxiety, working memory, and operation span, with *z* = 0 representing the sample mean. Figure 1 illustrates the standardized scores of the four indicators across the four profiles, allowing comparison of the relative emotional and cognitive characteristics of each group.

Profile 1, labeled Low Anxiety–Average Cognition (*n* = 117, 21.31%), was characterized by markedly below-average reading anxiety (*z* = −1.10) and math anxiety (*z* = −1.30), along with slightly above-average working memory (*z* = 0.37) and near-average operation span (*z* = −0.07). This pattern suggests that individuals in this profile experienced relatively low levels of both domain-specific anxieties while maintaining generally average to slightly better cognitive functioning.

Profile 2, labeled High Anxiety–High Cognition (*n* = 295, 53.73%), showed above-average reading anxiety (*z* = 0.35) and math anxiety (*z* = 0.44), together with above-average working memory (*z* = 0.52) and slightly above-average operation span (*z* = 0.17). This profile indicates that higher anxiety did not necessarily coincide with poorer cognitive performance. Instead, this group appeared to maintain relatively strong cognitive functioning despite elevated anxiety.

Profile 3, labeled Very Low Verbal Working Memory (*n* = 57, 10.38%), showed near-average reading anxiety (*z* = −0.07) and math anxiety (*z* = 0.00), slightly below-average operation span (*z* = −0.19), but extremely low working memory (*z* = −2.36). This pattern suggests that the defining feature of this profile was a substantial deficit in working memory rather than heightened anxiety. Compared with the other profiles, this group appeared to represent a distinct cognitive-risk subgroup.

Profile 4, labeled High Anxiety–Low Cognition (*n* = 80, 14.57%), was characterized by above-average reading anxiety (*z* = 0.43) and math anxiety (*z* = 0.34), together with below-average working memory (*z* = −0.66) and operation span (*z* = −0.36). This profile reflects a combined pattern of elevated anxiety and relatively weaker cognitive functioning, suggesting that emotional risk and cognitive vulnerability co-occurred in this group.

Overall, the four-profile solution suggested meaningful heterogeneity in the joint distribution of academic anxiety and cognitive performance. In particular, two profiles showed elevated academic anxiety but differed in cognitive functioning: one profile showed relatively strong cognitive performance, whereas the other showed lower working memory and operation span. This pattern supports a cautious interpretation that academic anxiety may be associated with more than one cognitive configuration, rather than a single uniform pattern of reduced working memory performance.

#### 3.1.2. Chinese-ARHQ Scores Across Profiles

Corrected Chinese-ARHQ scores were compared across the four latent profiles to examine whether the profiles differed in retrospective reading-difficulty history. The one-way ANOVA showed no significant profile differences, F(3, 546) = 0.285, *p* = 0.837, eta-squared = 0.002. Thus, within this sample, the emotion-cognition profiles did not appear to be closely indexed by ARHQ severity. This result should be interpreted cautiously: it does not imply that dyslexia-related history is unrelated to academic anxiety or cognition, but it suggests that ARHQ score alone did not distinguish the specific profiles identified in the present LPA.

#### 3.1.3. Sensitivity Analysis in the Elevated Dyslexia-Related Risk Subsample

The LPA was repeated in the elevated dyslexia-related risk subsample, defined as participants with corrected Chinese-ARHQ scores above 0.36. This subsample included 500 participants. The resulting four-profile solution was broadly consistent with the full-sample solution, including Low Anxiety-Average Cognition, High Anxiety-High Cognition, Very Low Verbal Working Memory, and High Anxiety-Low Cognition profiles (Table 4). This pattern suggests that the full-sample profile structure was not solely a consequence of including participants below the Chinese-ARHQ cutoff. Because the subsample substantially overlapped with the full sample, the analysis should be viewed as a within-sample sensitivity analysis rather than as independent validation.

## 4. Discussion

The present study used a person-centered approach to examine academic anxiety and working memory profiles in undergraduate students with varying levels of dyslexia-related risk. Using reading anxiety, math anxiety, verbal working memory, and operation span as profile indicators, the latent profile analysis identified four distinct emotion-cognition profiles: Low Anxiety-Average Cognition, High Anxiety-High Cognition, Very Low Verbal Working Memory, and High Anxiety-Low Cognition. These findings indicate that academic anxiety and cognitive functioning are not organized in a single uniform pattern. In particular, elevated reading and math anxiety did not necessarily correspond to poorer working memory performance. Instead, some individuals showed high academic anxiety together with relatively preserved cognitive functioning, whereas others showed high anxiety accompanied by weaker working memory and operation span performance.

The coexistence of the High Anxiety-High Cognition and High Anxiety-Low Cognition profiles also suggests that elevated dyslexia-related risk may be associated with heterogeneous emotion-cognition patterns rather than a single anxiety-related working-memory deficit. For individuals in the High Anxiety-Low Cognition profile, elevated academic anxiety may place additional demands on already limited working-memory resources, making them more vulnerable to difficulties during cognitively demanding academic tasks. This interpretation is consistent with evidence that, among university students with SLD, the negative association between state anxiety and academic performance was evident mainly when working memory was relatively low, but not when working memory was relatively high (Wang et al., 2025). In this context, state anxiety refers to transient, situation-specific anxiety experienced in academic contexts rather than a stable tendency. In contrast, individuals in the High Anxiety-High Cognition profile may have maintained relatively strong working-memory performance despite elevated anxiety, possibly because of stronger baseline cognitive resources, compensatory effort, or more effective learning strategies (Eysenck et al., 2007). Their elevated dyslexia-related risk may therefore reflect other reading- or language-related difficulties not captured by the working-memory indicators used in the present study, such as verbal comprehension, phonological processing, rapid naming, orthographic processing, or reading fluency (Hatcher et al., 2002; Tops et al., 2012). In addition, because dyslexia-related risk was assessed using a retrospective self-report questionnaire, current anxiety and negative academic self-perception may have influenced participants’ reports of their reading history. Thus, it may reflect different combinations of emotional burden, compensatory cognitive resources, unmeasured reading-related weaknesses, and retrospective self-report processes.

The Low Anxiety-Average Cognition profile may represent another non-anxiety-dominant pattern of dyslexia-related risk. Participants in this profile reported relatively low academic anxiety and showed generally average cognitive performance, suggesting that their dyslexia-related risk may not be primarily explained by current reading or mathematics anxiety, nor by marked working-memory weakness. One possible explanation is that some individuals may have experienced earlier reading-related difficulties but have developed compensatory strategies that allow them to maintain relatively stable cognitive and emotional functioning in adulthood (Faísca et al., 2023). Alternatively, their dyslexia-related risk may reflect reading- or language-specific weaknesses not captured by the present working-memory indicators, such as phonological processing, rapid naming, orthographic processing, reading fluency, verbal comprehension, or morphological awareness (Li et al., 2022). Because dyslexia-related risk was assessed using a retrospective self-report questionnaire, this profile may also partly reflect individuals’ remembered reading history rather than current impairment. Therefore, this profile should be interpreted cautiously as a possible compensated or non-anxiety-dominant risk pattern rather than as evidence that dyslexia-related risk is absent or fully explained by the measured anxiety and working-memory variables.

The Very Low Verbal Working Memory profile also suggests that cognitive vulnerability and emotional burden may be partially separable among adults with elevated dyslexia-related risk. This group was characterized primarily by very low digit-span working memory, whereas reading anxiety and mathematics anxiety were close to the sample average. This pattern does not strongly support an anxiety-driven explanation of dyslexia-related risk for all individuals (Smith-Spark & Fisk, 2007). At the same time, the profile should be interpreted carefully because digit-span performance may also be influenced by general factors such as task engagement, response validity, fatigue, and floor effects in a subset of participants. Although all participants were native Chinese speakers and reported no history of neurological disorders, very low digit-span scores may still reflect attention or concentration difficulties, processing speed differences, motivation, or task-specific floor effects rather than verbal working-memory limitations alone. Instead, it may reflect a cognitive-dominant pathway, in which verbal working-memory weakness is more salient than self-reported academic anxiety. For these individuals, dyslexia-related risk may be more closely associated with difficulties in maintaining, sequencing, or manipulating verbal information, which are important for reading-related processes (Trecy et al., 2013). However, because the present study was cross-sectional and relied on retrospective self-report to assess dyslexia-related risk, this profile should not be interpreted as evidence that weak verbal working memory is innate or directly causes dyslexia-related risk (He et al., 2025). Rather, it suggests that some adults with elevated dyslexia-related risk may show primary cognitive weaknesses that are not necessarily accompanied by high academic anxiety. At the same time, the profile should be interpreted carefully because digit-span performance may also be influenced by task engagement, response validity, or floor effects in a subset of participants.

Corrected Chinese-ARHQ scores did not significantly differ across the four profiles. This finding suggests that retrospective reading-difficulty severity, as measured by the ARHQ, was not the main feature distinguishing the current emotion-cognition profiles. The result does not mean that dyslexia-related history is unimportant. Rather, it indicates that students with comparable ARHQ-indexed risk may still differ in their current academic anxiety and working memory functioning. ARHQ screening may therefore be informative when combined with measures of current anxiety, cognitive performance, and academic functioning.

The sensitivity analysis in the elevated-risk subsample, which comprised 500 participants (90.91% of the total sample), provided a within-sample sensitivity check for the four-profile solution rather than an independent validation. The elevated-risk subsample largely overlapped with the full sample; therefore, similarity in profile patterns was expected. The same general pattern emerged among participants scoring above the Chinese-ARHQ cutoff, suggesting that the profiles were not driven only by the lower-risk participants. However, because the elevated-risk subsample was drawn from the same dataset and included most of the full sample, this result should be interpreted as a within-sample sensitivity analysis rather than as replication (Romero et al., 2025). These findings suggest cautious practical implications. Students in the High Anxiety-High Cognition profile may benefit from support targeting anxiety management, academic self-efficacy, and emotion regulation, even when working memory performance appears relatively preserved (Abu Omar et al., 2024), such as brief cognitive reappraisal training, test-anxiety reduction techniques, and metacognitive strategy instruction. Students in the High Anxiety–Low Cognition profile may require support that addresses both emotional burden and cognitive demands through instructional designs that reduce cognitive load (Sweller et al., 2019), including step-by-step task decomposition, explicit instruction with guided prompts, worked examples illustrating solution procedures, chunked presentation of information, and reduction in concurrent processing demands during complex academic tasks (van Merriënboer & Sweller, 2005). Students in the Very Low Verbal Working Memory profile may require more targeted cognitive and reading support. Practical supports may include text-to-speech systems, visually block or segmented reading materials, guided worksheets with external memory cues, and structured note-taking templates designed to reduce verbal working-memory demands during reading and learning activities (Wood et al., 2017; Mayer & Moreno, 2003). These implications should be treated as preliminary because the study did not test intervention outcomes directly.

## 5. Limitations

Several limitations should be considered. First, the sample consisted of undergraduate students from a single university, which may limit generalizability to broader adult populations or to adults with clinically diagnosed dyslexia. The use of campus-based convenience sampling and recruitment wording focused on reading-ability risk may have increased participation among students with reading-related or academic difficulties. This possible self-selection may partly explain why over 90% of participants were classified as showing elevated dyslexia-related risk. Therefore, this proportion should be interpreted as a sample-specific screening result rather than as a prevalence estimate for university students aged 18–25. Second, the cognitive measures used in the present study were limited to digit span and operation span tasks, which primarily assess verbal working memory. Other important reading-related cognitive mechanisms, such as phonological awareness and rapid automatized naming (RAN), were not assessed. Therefore, the identified profiles should be interpreted as reflecting heterogeneity in academic anxiety and working-memory functioning rather than the full cognitive heterogeneity associated with dyslexia-related risk. Third, the cross-sectional design prevents causal inference. The data cannot determine whether anxiety influences working memory, weaker cognitive performance contributes to anxiety, or both are shaped by shared developmental or educational experiences. Fourth, the unusually high proportion of participants above the Chinese-ARHQ cutoff may partly reflect the specific recruitment context of a single regional undergraduate institution, rather than the prevalence of dyslexia-related risk in the broader undergraduate population. Therefore, the cutoff-based classification should be interpreted as a sample-specific screening indicator and should be validated in more diverse university samples. Fifth, latent profile membership is probabilistic and should not be treated as a clinical classification. Replication in independent samples is needed to determine whether the same profiles are stable across settings. Finally, the presence of very low or zero working memory scores for some participants raises the possibility of floor effects or response validity issues.

## 6. Conclusions

In conclusion, the present findings provide preliminary evidence that adults with varying dyslexia-related risk may show distinct configurations of academic anxiety and working memory functioning. Elevated reading and mathematics anxiety were not uniformly associated with poorer cognitive performance, and retrospective ARHQ scores did not significantly differentiate the identified profiles. These results support a more nuanced, person-centered view of academic anxiety and working memory in adults with dyslexia-related risk, while underscoring the need for replication with independent samples and more comprehensive reading and cognitive assessments.

## Figures and Tables

**Table 1:** Descriptive statistics and bivariate correlations for all focal variables.

Variable	Mean	SD	1	2	3	4	5	6
1. Mathematics Anxiety	64.165	20.214	1					
2. Reading Anxiety	30.696	9.782	0.564 ***	1				
3. ARHQ score	0.712	0.072	0.076 *	0.172 ***	1			
4. Forward WM	6.058	2.788	−0.001	−0.013	0.019	1		
5. Backward WM	7.440	3.022	−0.036	−0.054	0.047	0.659 ***	1	
6. Operation Span	9.031	3.715	−0.002	0.014	−0.045	0.127 **	0.183 ***	1

Note. *** *p* < 0.001, ** *p* < 0.01, * *p* < 0.05.

**Table 2:** Latent profile analysis fit indices for the emotion-cognition profiles.

	3 Profiles	4 Profiles	5 Profiles	6 Profiles
Number of free parameters	18	23	28	33
AIC	5777.85	5716.76	5678.67	5649.19
BIC	5855.40	5815.85	5799.30	5791.35
SSA-BIC	5798.26	5742.84	5710.41	5686.60
Entropy	0.85	0.83	0.85	0.82
VLMR-LRT *p*	<0.001	0.003	0.023	0.064
Profile sizes	121; 72; 356	117; 295; 57; 80	13; 47; 117; 80; 292	33; 106; 219; 56; 86; 49
Average posterior probabilities	0.887; 0.948; 0.940	0.888; 0.907; 0.991; 0.832	0.943; 0.932; 0.905; 0.848; 0.904	0.833; 0.829; 0.872; 0.994; 0.840; 0.891

Note. AIC = Akaike Information Criterion; BIC = Bayesian Information Criterion; SSA-BIC = sample-size adjusted Bayesian Information Criterion; VLMR-LRT = Vuong-Lo-Mendell-Rubin likelihood ratio test.

**Table 3:** Standardized indicator means for the four emotion-cognition profiles.

	Profile 1 Low Anxiety-Average Cognition *n* = 117 (21.31%)	Profile 2 High Anxiety-High Cognition *n* = 295 (53.73%)	Profile 3 Very Low Verbal Working Memory *n* = 57 (10.38%)	Profile 4 High Anxiety-Low Cognition *n* = 80 (14.57%)
Reading Anxiety	−1.10 (0.08)	0.35 (0.06)	−0.07 (0.12)	0.43 (0.14)
Mathematics Anxiety	−1.30 (0.08)	0.44 (0.06)	0.00 (0.13)	0.34 (0.11)
Verbal Working Memory	0.37 (0.06)	0.52 (0.04)	−2.36 (0.04)	−0.66 (0.11)
Operation Span	−0.07 (0.11)	0.17 (0.07)	−0.19 (0.14)	−0.36 (0.13)

Note. Scores are presented as mean (SE).

**Table 4:** Profile sizes and standardized indicator means for the four-profile solution in the elevated dyslexia-related risk subsample.

	Profile 1 Low Anxiety-Average Cognition *n* = 103 (20.64%)	Profile 2 High Anxiety-High Cognition *n* = 268 (53.71%)	Profile 3 Very Low Verbal Working Memory *n* = 54 (10.82%)	Profile 4 High Anxiety-Low Cognition *n* = 74 (14.83%)
Reading Anxiety	−1.13 (0.08)	0.35 (0.07)	0.01 (0.12)	0.35 (0.15)
Mathematics Anxiety	−1.34 (0.10)	0.43 (0.06)	0.10 (0.12)	0.32 (0.12)
Verbal Working Memory	0.37 (0.07)	0.52 (0.04)	−2.35 (0.04)	−0.62 (0.11)
Operation Span	−0.09 (0.12)	0.18 (0.07)	−0.18 (0.14)	−0.36 (0.13)

Note. Scores are presented as mean (SE).

## Data Availability

The original contributions presented in this study are included in the article or Supplementary Material. Further inquiries can be directed to the corresponding author(s).

## Supplementary Materials

The following supporting information can be downloaded at: https://www.mdpi.com/article/10.3390/jintelligence14070114/s1, Dataset S1: Dataset including variables and responses.

## References

[B1-jintelligence-14-00114] Abu Omar D., Kirkman A., Scott C., Babicova I., Irons Y. (2024). Positive psychology interventions to increase self-esteem, self-efficacy, and confidence and decrease anxiety among students with dyslexia: A narrative review. Youth.

[B2-jintelligence-14-00114] Ashcraft M. H., Krause J. A. (2007). Working memory, math performance, and math anxiety. Psychonomic Bulletin & Review.

[B3-jintelligence-14-00114] Broks V. M. A., Dijk S. W., Van den Broek W. W., Stegers-Jager K. M., Woltman A. M. (2024). Self-regulated learning profiles including test anxiety linked to stress and performance: A latent profile analysis based across multiple cohorts. Medical Education.

[B4-jintelligence-14-00114] Brumariu L. E., Waslin S. M., Gastelle M., Kochendorfer L. B., Kerns K. A. (2023). Anxiety, academic achievement, and academic self-concept: Meta-analytic syntheses of their relations across developmental periods. Development and Psychopathology.

[B5-jintelligence-14-00114] Carroll J. M., Iles J. E. (2006). An assessment of anxiety levels in dyslexic students in higher education. British Journal of Educational Psychology.

[B6-jintelligence-14-00114] Chow B. W.-Y., Mo J., Dong Y. (2021). Roles of reading anxiety and working memory in reading comprehension in English as a second language. Learning and Individual Differences.

[B7-jintelligence-14-00114] Dowker A., Sarkar A., Looi C. Y. (2016). Mathematics anxiety: What have we learned in 60 years?. Frontiers in Psychology.

[B8-jintelligence-14-00114] Eckert L., Hartwigsen G., Turker S. (2025). Cognitive–linguistic profiles of German adults with dyslexia. Behavioral Sciences.

[B9-jintelligence-14-00114] Edwards A. A., Daucourt M. C., Hart S. A., Schatschneider C. (2023). Measuring reading anxiety in college students. Reading and Writing.

[B10-jintelligence-14-00114] Enders C. K. (2001). The performance of the full information maximum likelihood estimator in multiple regression models with missing data. Educational and Psychological Measurement.

[B11-jintelligence-14-00114] Eysenck M., Derakshan N., Santos R., Calvo M. (2007). Anxiety and cognitive performance: Attentional control theory. Emotion.

[B12-jintelligence-14-00114] Faísca L., Reis A., Araújo S. (2023). Cognitive subtyping of university students with dyslexia in a semi-transparent orthography: What can weaknesses and strengths tell us about compensation?. Journal of Cultural Cognitive Science.

[B13-jintelligence-14-00114] Francis D. A., Caruana N., Hudson J. L., McArthur G. M. (2019). The association between poor reading and internalising problems: A systematic review and meta-analysis. Clinical Psychology Review.

[B14-jintelligence-14-00114] Hatcher J., Snowling M. J., Griffiths Y. M. (2002). Cognitive assessment of dyslexic students in higher education. British Journal of Educational Psychology.

[B15-jintelligence-14-00114] He Y., Tang J., Yang X., Song Z., Ding N., Jia Y., Liu L., Zhao J. (2025). Development and validity of the adult reading history questionnaire (ARHQ) for Chinese. Dyslexia.

[B16-jintelligence-14-00114] Jaffe J., Bolger D. (2023). Cognitive processes, linguistic factors, and arithmetic word problem success: A review of behavioral studies. Educational Psychology Review.

[B17-jintelligence-14-00114] Jordan J., McGladdery G., Dyer K. (2014). Dyslexia in higher education: Implications for maths anxiety, statistics anxiety and psychological well-being. Dyslexia.

[B18-jintelligence-14-00114] Lai Y., Zhu X., Chen Y., Li Y. (2015). Effects of mathematics anxiety and mathematical metacognition on word problem solving in children with and without mathematical learning difficulties. PLoS ONE.

[B19-jintelligence-14-00114] Lefly D. L., Pennington B. F. (2000). Reliability and validity of the adult reading history questionnaire. Journal of Learning Disabilities.

[B20-jintelligence-14-00114] Li X., Hu M., Liang H. (2022). The percentages of cognitive skills deficits among Chinese children with developmental dyslexia: A systematic review and meta-analysis. Brain Sciences.

[B21-jintelligence-14-00114] Matt C. H., Michael E. H. (2018). Variable-centered, person-centered, and person-specific approaches. Organizational Research Methods.

[B22-jintelligence-14-00114] Mayer R. E., Moreno R. (2003). Nine ways to reduce cognitive load in multimedia learning. Educational Psychologist.

[B23-jintelligence-14-00114] Moojen S. M. P., Gonçalves H. A., Bassôa A., Navas A. L., de Jou G., Miguel E. S. (2020). Adults with dyslexia: How can they achieve academic success despite impairments in basic reading and writing abilities? The role of text structure sensitivity as a compensatory skill. Annals of Dyslexia.

[B24-jintelligence-14-00114] Moran T. P. (2016). Anxiety and working memory capacity: A meta-analysis and narrative review. Psychological Bulletin.

[B25-jintelligence-14-00114] Nylund K. L., Asparouhov T., Muthén B. O. (2007). Deciding on the number of classes in latent class analysis and growth mixture modeling: A Monte Carlo simulation study. Structural Equation Modeling: A Multidisciplinary Journal.

[B26-jintelligence-14-00114] Ostrosky-Solís F., Lozano A. (2006). Digit span: Effect of education and culture. International Journal of Psychology.

[B27-jintelligence-14-00114] Peng P., Barnes M., Wang C., Wang W., Li S., Swanson H., Dardick W., Tao S. (2018). A meta-analysis on the relation between reading and working memory. Psychological Bulletin.

[B28-jintelligence-14-00114] Reis A., Araújo S., Morais I. S., Faísca L. (2020). Reading and reading-related skills in adults with dyslexia from different orthographic systems: A review and meta-analysis. Annals of Dyslexia.

[B29-jintelligence-14-00114] Romero L. S., O’Malley M., Carter D. (2025). Replication using confirmatory latent class analysis: A school climate example. Journal of School Psychology.

[B30-jintelligence-14-00114] Sasanguie D., Larmuseau C., Depaepe F., Jansen B. R. J. (2024). Anxiety about mathematics and reading in preadolescents is domain-specific. Journal of Intelligence.

[B31-jintelligence-14-00114] Smith-Spark J. H., Fisk J. E. (2007). Working memory functioning in developmental dyslexia. Memory.

[B32-jintelligence-14-00114] Snowling M. J., Hulme C., Nation K. (2020). Defining and understanding dyslexia: Past, present and future. Oxford Review of Education.

[B33-jintelligence-14-00114] Suinn R. M., Edie C. A., Nicoletti J., Spinelli P. R. (1972). The MARS, a measure of mathematics anxiety: Psychometric data. Journal of Clinical Psychology.

[B34-jintelligence-14-00114] Suinn R. M., Edwards R. (1982). The measurement of mathematics anxiety: The mathematics anxiety rating scale for adolescents—MARS-A. Journal of Clinical Psychology.

[B35-jintelligence-14-00114] Sweller J., van Merriënboer J. J. G., Paas F. (2019). Cognitive architecture and instructional design: 20 years later. Educational Psychology Review.

[B36-jintelligence-14-00114] Tal D., Dotan S., Ben-Yair M., Katzir T., Rubinsten O. (2025). Arithmetic and reading skills mediate the link between math and reading anxiety and word problem solving. Scientific Reports.

[B37-jintelligence-14-00114] Tein J., Coxe S., Cham H. (2013). Statistical power to detect the correct number of classes in latent profile analysis. Structural Equation Modeling: A Multidisciplinary Journal.

[B38-jintelligence-14-00114] Tops W., Callens M., Lammertyn J., Van Hees V., Brysbaert M. (2012). Identifying students with dyslexia in higher education. Annals of Dyslexia.

[B39-jintelligence-14-00114] Trecy M. P., Steve M., Martine P. (2013). Impaired short-term memory for order in adults with dyslexia. Research in Developmental Disabilities.

[B40-jintelligence-14-00114] Turner M. L., Engle R. W. (1989). Is working memory capacity task dependent?. Journal of Memory and Language.

[B41-jintelligence-14-00114] Unsworth N., Heitz R. P., Schrock J. C., Engle R. W. (2005). An automated version of the operation span task. Behavior Research Methods.

[B42-jintelligence-14-00114] Valentine T., Block C., Eversole K., Boxley L., Dawson E. (2020). Wechsler adult intelligence scale-IV (WAIS-IV). The Wiley encyclopedia of personality and individual differences: Clinical, applied, and cross-cultural research.

[B43-jintelligence-14-00114] van Merriënboer J. J. G., Sweller J. (2005). Cognitive load theory and complex learning: Recent developments and future directions. Educational Psychology Review.

[B44-jintelligence-14-00114] Vieira A. P. A., Peng P., Antoniuk A., DeVries J., Rothou K., Parrila R., Georgiou G. (2024). Internalizing problems in individuals with reading, mathematics and unspecified learning difficulties: A systematic review and meta-analysis. Annals of Dyslexia.

[B45-jintelligence-14-00114] Wang L.-C., Chung K. K.-H., Jhuo R.-A. (2025). The relationships among working memory, state anxiety, and academic performance in Chinese undergraduates with SLD. Reading and Writing.

[B46-jintelligence-14-00114] Welcome S. E., Meza R. A. (2019). Dimensions of the adult reading history questionnaire and their relationships with reading ability. Reading and Writing.

[B47-jintelligence-14-00114] Wood S. G., Moxley J. H., Tighe E. L., Wagner R. K. (2017). Does use of text-to-speech and related read-aloud tools improve reading comprehension for students with reading disabilities? A meta-analysis. Journal of Learning Disabilities.

[B48-jintelligence-14-00114] Xiao P., Zhu K., Liu Q., Xie X., Jiang Q., Feng Y., Wu X., Tang J., Song R. (2022). Association between developmental dyslexia and anxiety/depressive symptoms among children in China: The chain mediating of time spent on homework and stress. Journal of Affective Disorders.

